# Geo-mapping of caries risk in children and adolescents - a novel approach for allocation of preventive care

**DOI:** 10.1186/1472-6831-11-26

**Published:** 2011-09-26

**Authors:** Ulf Strömberg, Kerstin Magnusson, Anders Holmén, Svante Twetman

**Affiliations:** 1 Department of Research and Development, Halland Hospital, SE-301 85 Halmstad, Sweden; 2 Department of Occupational and Environmental Medicine, Lund University, SE-221 85 Lund, Sweden; 3 Section of Community and Preventive Dentistry, Maxillofacial Unit, Halland Hospital, SE-301 85 Halmstad, Sweden; 4 Department of Cariology, Endodontics and Pediatric Dentistry, Institute of Dentistry, Faculty of Health Sciences, University of Copenhagen, Nørre Allé 20, 2200 Copenhagen N, Denmark

**Keywords:** caries, children, prevention, geo-mapping

## Abstract

**Background:**

Dental caries in children is unevenly distributed within populations with a higher burden in low socio-economy groups. Thus, tools are needed to allocate resources and establish evidence-based programs that meet the needs of those at risk. The aim of the study was to apply a novel concept for presenting epidemiological data based on caries risk in the region of Halland in southwest Sweden, using geo-maps.

**Methods:**

The study population consisted of 46,536 individuals between 3-19 years of age (75% of the eligible population) from whom caries data were reported in 2010. Reported dmfs/DMFS>0 for an individual was considered as the primary caries outcome. Each study individual was geo-coded with respect to his/her residence parish. A parish-specific relative risk (RR) was calculated as the observed-to-expected ratio, where the expected number of individuals with dmfs/DMFS>0 was obtained from the age- and sex-specific caries (dmfs/DMFS>0) rates for the total study population. Smoothed caries risk geo-maps, along with corresponding statistical certainty geo-maps, were produced by using the free software Rapid Inquiry Facility and the ESRI^® ^ArcGIS system.

**Results:**

The geo-maps of preschool children (3-6 years), schoolchildren (7-11 years) and adolescents (12-19 years) displayed obvious geographical variations in caries risk, albeit most marked among the preschoolers. Among the preschool children the smoothed relative risk (SmRR) varied from 0.33 to 2.37 in different parishes. With increasing age, the contrasts seemed to diminish although the gross geographical risk pattern persisted also among the adolescents (SmRR range 0.75-1.20).

**Conclusion:**

Geo-maps based on caries risk may provide a novel option to allocate resources and tailor supportive and preventive measures within regions with sections of the population with relatively high caries rates.

## Background

In spite of the global decline in childhood caries, widening inequalities in oral health exist between social classes and certain minority ethnic groups [1,2]. In the United Kingdom, this has primarily been observed among preschool- and schoolchildren [3,4]. Also in Scandinavia, where almost all children and adolescents attend the prevention-oriented free public dental service, a social gradient for dental health is evident [5-7]. In order to reduce these gaps, various oral health promotion activities has been suggested and, preferable, integrated with general health education since oral diseases and chronic systemic diseases share many common risk factors [8,9]. The challenge and crucial decision for policymakers and professionals are to allocate resources and establish evidence-based programs that meet the needs of the vulnerable children at risk for caries. The allocation is traditionally based on conventional caries epidemiological data in spite of the fact that the children by then already are diseased. Oral health programs based on caries risk would be more proactive since a comprehensive risk assessment is an essential component in the decision-making process for the prevention and management of dental caries [10]. Thus, the aims of the present communication were to suggest a novel approach to present epidemiological data based on caries risk in a population. We launch the use of geo-maps and apply the concept in the southwest Swedish region of Halland.

## Methods

### Study population

The Halland region has approximately 70,000 inhabitants below the age of 20 years and the vast majority is listed as regular patients at the Public Dental Service that provides free dental care between 1 and 19 years with recall intervals varying from 3 to 24 months depending on the individual need. Data on the experience of manifest (dentin) caries is registered according to the WHO-criteria [11] and annually reported to the community dentistry unit. The present study population included 46,536 individuals for whom caries data were reported in year 2010; they were between 3-19 years old when examined. The overall coverage was 75% of the total 3-19-year population of the region. The remaining children were not recalled for a regular check-up that year or visited a private dentist outside the region. In Halland, the fluoride concentration in piped water supply is low (<0.3 ppm) except in the northern part (the municipality of Kungsbacka) where the natural fluoride content is approximately 1.0 ppm. There is also a geographical variation in socio-economic characteristics of the population in the Halland region. For example, the proportion with post-secondary education among all residents varies between 10 to 48% across different parishes (data from year 2010 provided from Statistics Sweden). The study was approved by the Halland Hospital Ethical committee as well as The Swedish Data Inspection Board.

### Geographical information system (GIS) methods

**Figure 1:**
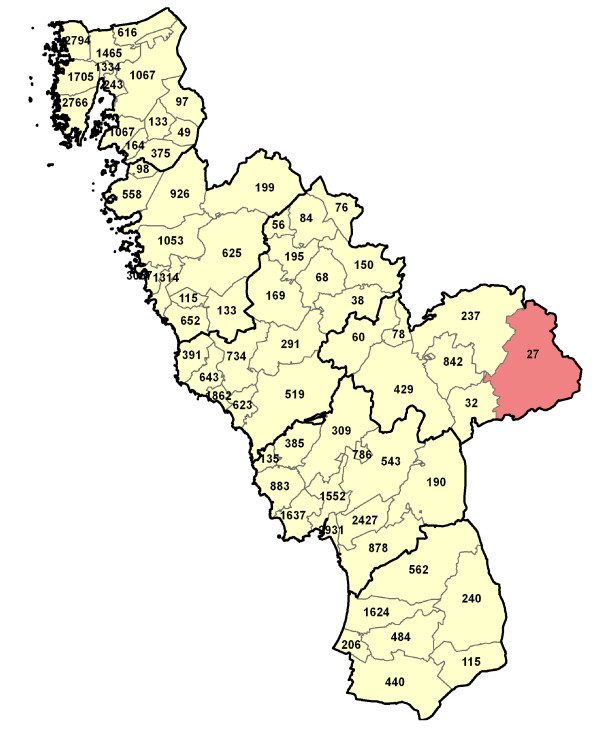
**Distribution of participants**. Geo-map of the Halland region (southwest Sweden) showing the number of study person between 3-19 years of age in each of the 66 residential parishes. The thicker borderlines delimit the six municipalities of Halland. The coverage (i.e., proportion of the eligible population) varied between 61-89% in the different parishes, except in one parish (red background) where the coverage was only 12%.

The Halland region consists of six municipalities that are subdivided into 66 parishes. Geo-maps were produced by using the ESRI^® ^ArcGIS system (Environmental Systems Research Institute, Inc., USA). Each study individual was geo-coded with respect to his/her residence area (parish). Figure 1 shows the number of study persons in each parish

### Epidemiological and statistical methods

Reported dmfs/DMFS >0 for an individual was considered as the primary caries outcome. A parish-specific relative risk (RR) was calculated as the observed-to-expected ratio, where the expected number of individuals with dmfs/DMFS >0 was obtained from the age- and sex-specific caries (dmfs/DMFS >0) rates for the whole region of Halland or, more precisely, for the total study population. The following age strata were used: 3-6, 7-11,12-18, and 19 years. Thus, the expected number for a parish equals the sum of the products *n_i_*×*r_i _*across the age- and sex-strata *i*(3-6 year old girls; 3-6 year old boys; 7-11 year old girls; etc.), where *n_i _*denotes the stratum-specific number of study individuals residing in the parish and *r_i _*denotes the corresponding caries rate observed in the total study population. The computations of the RRs were performed using the free software Rapid Inquiry Facility [12], which provides an extension to ESRI^® ^ArcGIS functions [13]. The Rapid Inquiry Facility (RIF) along with free software for Bayesian data analyses, WinBUGS [14], provides a powerful tool for geo-mapping based on epidemiological data. The caries risk maps show the smoothed RRs (SmRR) for each parish, which were obtained by running the Bayesian hierarchical mapping model in RIF/WinBUGS. We underline that such Bayesian smoothing yields pronounced downward adjustment of a (conventional) RR for a parish with few study persons, estimated with relatively high uncertainty, if that RR turns out notably elevated. Hence, by presenting smoothed caries risk geo-maps, rational adjustments of the conventional (parish-specific) RRs are taken into account [15,16].

We present separate caries risk geo-maps for preschool children (3-6 years), schoolchildren (7-11 years) and adolescents [12-19 years; based on age-stratified (12-18 and 19 years, respectively) analysis]. Along with each caries risk geo-map, we provide the corresponding statistical certainty geo-map. A posterior probability of a parish-specific relative risk above one given the data, denoted Pr(RR>1|data), was obtained by the Bayesian approach. A parish with data yielding strong statistical evidence of an elevated caries risk, more precisely Pr(RR>1|data) > 0.95, was colored red in the certainty geo-map. By contrast, a parish with evidently low caries risk, Pr(RR<1|data) > 0.95, was colored green. Analogously, parish-specific 90% credibility intervals for the relative risk were obtained; and each parish with a 90% credibility interval that covers 1 was colored yellow in the certainty geo-map indicating a weaker statistical evidence for a high or low relative risk in such parishes.

We addressed geographical co-variations between caries risk and residents'' level of education by calculating Spearman''s correlations (r_S_) between the SmRRs and the proportions with post-secondary educational level among the residents (considered as a group-level indicator of socio-economy) across the 66 parishes.

## Results

**Figure 2:**
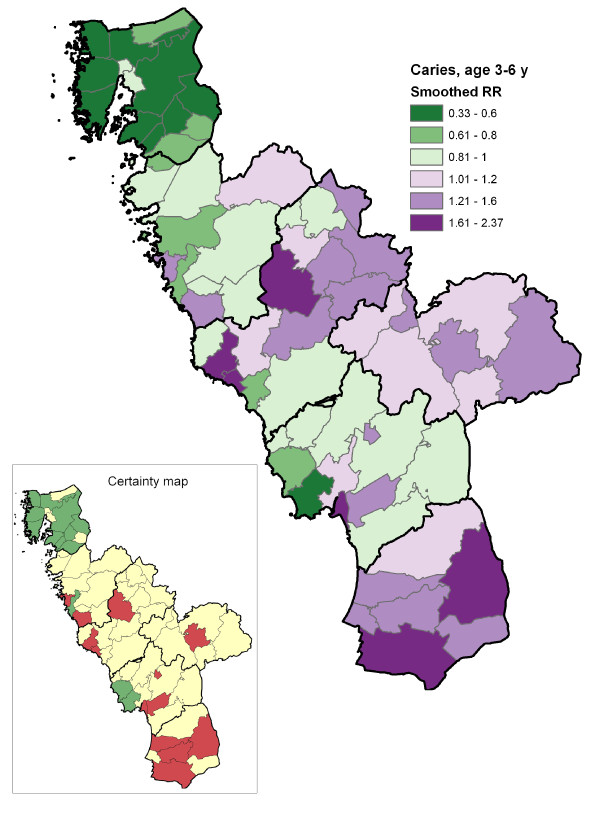
**Geo-map of caries risk in preschool children**. Caries risk geo-map of the Halland region (southwest Sweden) displaying, for each of the 66 residential parishes, the smoothed relative risk (SmRR, range between 0.33-2.37) of caries (dmfs/DMFS >0) among preschoolers (3-6 years). The thicker borderlines delimit the six municipalities of Halland. The corresponding statistical certainty geo-map is also shown [*red color*, Pr(RR>1|data) > 0.95, i.e. a parish with data yielding strong statistical evidence of an elevated caries risk; *green color*, Pr(RR<1|data) > 0.95, i.e. a parish with data yielding strong statistical evidence of a low caries risk; and *yellow color*, the 90% credibility interval covers RR = 1, i.e. a parish with data yielding weaker statistical evidence for a high or low relative risk].

**Figure 3:**
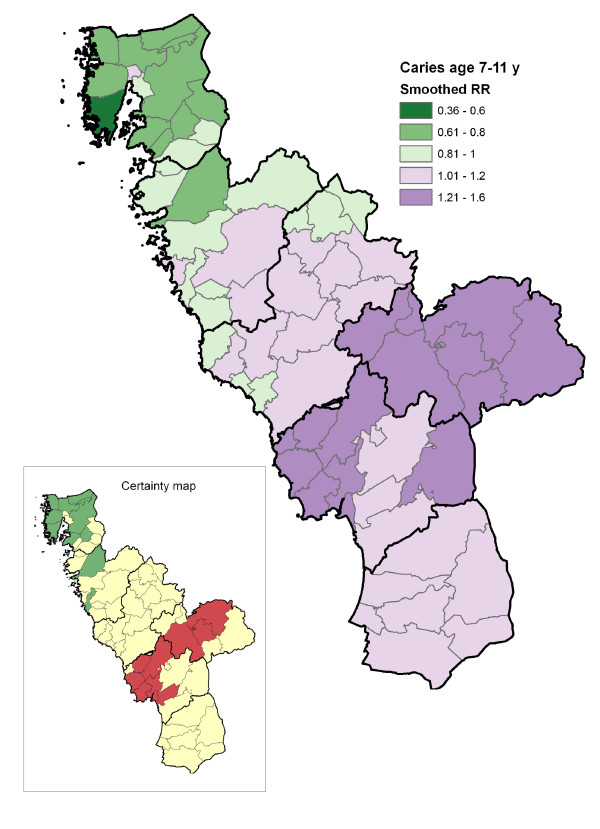
**Geo-map of caries risk in schoolchildren**. Caries risk geo-map of the Halland region (southwest Sweden) displaying, for each of the 66 residential parishes, the smoothed relative risk (SmRR, range between 0.36-1.47) of caries (dmfs/DMFS >0) among schoolchildren (7-11 years). The thicker borderlines delimit the six municipalities of Halland. The corresponding statistical certainty geo-map is also shown [*red color*, Pr(RR>1|data) > 0.95, i.e. a parish with data yielding strong statistical evidence of an elevated caries risk; *green color*, Pr(RR<1|data) > 0.95, i.e. a parish with data yielding strong statistical evidence of a low caries risk; and *yellow color*, the 90% credibility interval covers RR = 1, i.e. a parish with data yielding weaker statistical evidence for a high or low relative risk].

**Figure 4:**
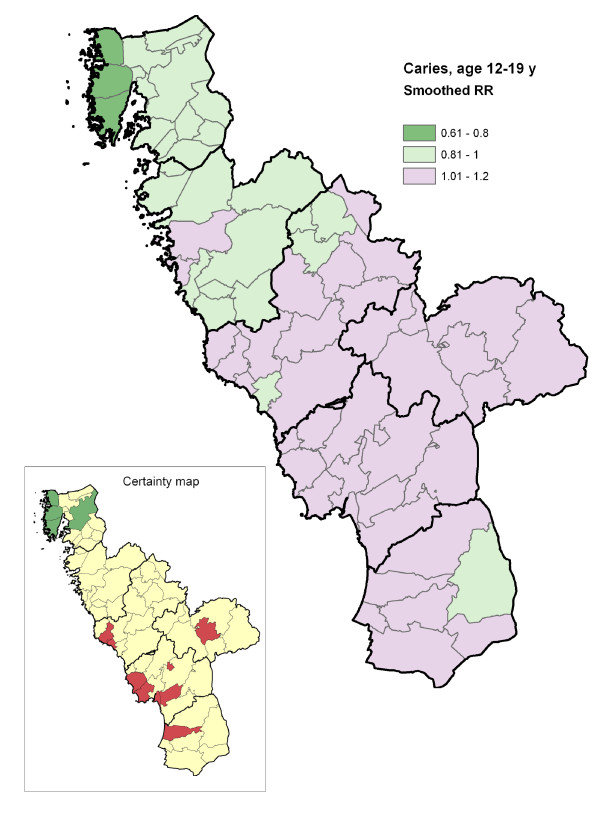
**Geo-map of caries risk in adolescents**. Caries risk geo-map of the Halland region (southwest Sweden) displaying, for each of the 66 residential parishes, the smoothed relative risk (SmRR, range between 0.75-1.20) of caries (dmfs/DMFS >0) among adolescents (12-19 years). The thicker borderlines delimit the six municipalities of Halland. The corresponding statistical certainty geo-map is also shown [*red color*, Pr(RR>1|data) > 0.95, i.e. a parish with data yielding strong statistical evidence of an elevated caries risk; *green color*, Pr(RR<1|data) > 0.95, i.e. a parish with data yielding strong statistical evidence of a low caries risk; and *yellow color*, the 90% credibility interval covers RR = 1, i.e. a parish with data yielding weaker statistical evidence for a high or low relative risk].

The proportion of children with no obvious decay at various ages and the cumulative burden of caries in the region, expressed as mean dmfs/DMFS, are shown in Table 1. The geo-maps of caries risk for preschool children, schoolchildren and adolescents are presented in Figures 2, 3 and 4. The geographical variation in caries risk was obvious. Among the preschool children, the smoothed relative risk (SmRR) varied from 0.33 to 2.37 in different parishes. With increasing age, the contrasts seemed to diminish although the gross geographical risk pattern persisted also among the adolescents (SmRR range 0.75-1.20). As expected, the lowest caries risk (dark green) was seen for all ages in the northern area with the elevated natural fluoride content in water supply. The across-parish-correlation between the SmRRs and the proportions with post-secondary educational level among the residents was highly significant for preschool children (r_S _= -0.59; p < 0.01), but not for schoolchildren (r_S _= -0.21; p = 0.10) and adolescents (r_S _= -0.12; p = 0.24).

**Table 1:** Prevalence and experience of manifest (dentin) caries on surface level in the Region of Halland 2010

Age group	Dmfs/DMFS = 0	Mean dmfs/DMFS (95% CI)
3-6 yr	88.40	0.59* (0.54-0.64)
7-11 yr	84.04	0.34§ (0.32-0.335)
12-18 yr	47.23	2.20 (2.14-2.25)
19 yr	28.52	4.10 (3.92-4.28)

Children with no obvious decay was defined as dmfs/DMFS = 0

*primary teeth only

§permanent teeth only

## Discussion

It is generally recognized that GIS may play an essential role in helping public health organizations understand population health and make decisions [17]. The methods we applied have previously been utilized, e.g. within general medicine for risk mapping of common chronic diseases [18]. However, dental caries is also a common chronic disease and it is evident that severe caries in childhood affects the quality of life. It is also clear that the skew burden of the disease calls for allocation of resources and manpower for preventive activities among those with the highest need. To our knowledge, the GIS concept has not previously been used within dentistry in the present manner. We have noticed close similarities with geo-maps based on some other diseases and medical conditions with life-style and behavior-related determinants, which is interesting. Thus, by addressing the common risk factor approach (high-sugar diet, fats, smoking, alcohol, lack of control, etc), dental professionals will not only prevent dental diseases but will also contribute to preventing obesity, heart disease and diabetes [19]. The co-morbidity of the chronic dental and medical conditions is strong argument for the allocation of monetary and human resources according to the "directed vulnerable population strategy" (DVPS) that may be cost saving in the long perspective [20].

From an international perspective, it should be pointed out that the overall cumulative caries burden in the present study population was very low (Table 1) [21]. Although the social and fluoride gradients on caries risk were quite expected, the produced geo-maps certainly provided some interesting and useful information for oral health planners. Educational level was considered a valid, objective socioeconomic indicator in this study area; it is also a parish-level measure that has been stable during recent years. In general, the educational level is higher in the urban residential areas and lower in the rural areas. Within the urban areas (located along coastline in the west) the social gradient could be explained by other factors such as family income and proportion of immigrants. The social gradient could be elaborated more in detail. Additional data on contextual/geographic and individual variables that are related to the caries outcome could allow for more extensive multilevel modeling. Nevertheless, investigators should consider adjustments for contextual and individual predictors with caution, depending on their purpose being to disclose vulnerable groups or provide further insights regarding the underlying predictors. Indeed, a quick glance at the present geo-maps could assist in making policies for reducing dental caries in vulnerable groups. For example, in the centre of the low-risk high-fluoride Kungsbacka municipality in the northern part of the region, an urban parish (1,334 study persons) with a notably higher caries risk among pre- and schoolchildren was identified (Figures 2 and 3). Schools and nurseries are excellent arenas to promote a healthy lifestyle and self-care practices in children [19]. Therefore, a local school-based fluoride and healthy-habit activity could be implemented in such a parish; fluorides have been proven to be effective in controlling and preventing caries and are a vital component of all caries prevention programs [22-24].

Another interesting example was the demonstrated age-related impact of the smoothed relative risk which may imply that a relatively larger proportion of the preventive efforts should be steered towards the youngest age groups. Among the preschoolers, the SmRRs captured the geographical risk pattern of early childhood caries. There are several documented examples of successful programs for infant feeding and early fluoride exposure that have reduced inequalities in oral health in preschool children in a cost effective way [25]. In the light of the examples given above, the suggested geo-maps may be an additional tool for allocation of necessary resources to dental practitioners to act as health advocates and monitor out-reach interventions performed by auxiliary staff. Among the adolescents, who have pronounced cumulative caries burden (Table 1), complementary measures to the SmRR (considering also mean dmfs/DMS) might be taken into account as well, in order to better capturing the geographical contrasts in caries burden.

## Conclusion

In summary, geo-maps based on caries risk may provide a novel option to allocate resources and tailor supportive and preventive measures within regions with sections of the population with relatively high caries rates.

## Competing interests

The authors declare that they have no competing interests.

## Authors' contributions

All of the listed authors contributed to the conduct of the study. US and ST analyzed and interpreted the data and drafted the manuscript. KE provided technical and administrative support and AH performed the computer work. All authors approved the final version of this manuscript.

## Pre-publication history

The pre-publication history for this paper can be accessed here:

http://www.biomedcentral.com/1472-6831/11/26/prepub
